# Clinical Predictors of Response to Testosterone Replacement Therapy in Boys With Micropenis: A Retrospective Study Focusing on Penile Morphology and Hormonal Factors

**DOI:** 10.1111/iju.70204

**Published:** 2025-08-14

**Authors:** Junki Harada, Kensuke Mitsunari, Shota Kakita, Hajime Fukushima, Haruka Kawamura, Hiroki Kurata, Tsuyoshi Matsuda, Midori Motokawa, Itsuho Ito, Kyohei Araki, Yuichiro Nakamura, Tomohiro Matsuo, Sumito Dateki, Toshiharu Kihara, Kojiro Ohba, Yasushi Mochizuki, Ryoichi Imamura

**Affiliations:** ^1^ Department of Urology Nagasaki University Graduate School of Biomedical Sciences Nagasaki Japan; ^2^ Department of Pediatrics Nagasaki University Hospital Nagasaki Japan; ^3^ Department of Pediatrics Saiseikai Nagasaki Hospital Nagasaki Japan; ^4^ Department of Urology Nagasaki Memorial Hospital Nagasaki Japan

**Keywords:** glans width, micropenis, mini‐puberty, stretched penile length, testosterone replacement therapy

## Abstract

**Objectives:**

We aimed to identify clinical predictors of response to testosterone replacement therapy in boys with micropenis, focusing on initial penile measurements, hormone levels, and treatment timing relative to the mini‐puberty period.

**Methods:**

This retrospective study included 37 boys aged ≤ 3 years with micropenis who received three intramuscular injections of testosterone enanthate (25 mg at monthly intervals) at Nagasaki University Hospital between April 2019 and March 2024. Based on post‐treatment stretched penile length standard deviation scores, the patients were classified into three response groups: good (Group 1), intermediate (Group 2), and poor (Group 3). Baseline pre‐treatment stretched penile length, glans width, hormone levels, and timing of testosterone replacement therapy were compared among the groups. Logistic regression analysis was used for identifying predictors of favorable response.

**Results:**

There were statistically significant differences in stretched penile length and glans width among the three groups. Although testosterone replacement therapy initiated during mini‐puberty was more frequent in Group 3 than in the other groups, this difference was not substantial. Using logistic regression analysis, we identified baseline pretreatment stretched penile length and glans width as independent predictors of treatment response, with glans width showing stronger predictive power.

**Conclusion:**

Initial penile dimensions, particularly glans width, were reliable predictors of testosterone replacement therapy efficacy in boys with micropenis. Further prospective studies are warranted to optimize treatment timing and assess underlying hormonal and genetic factors.

**Abbreviations**
AR: androgen receptor
CI: confidence interval
DHT: dihydrotestosterone
FSH: follicle‐stimulating hormone
GR: growth ratio
HPG: hypothalamicーpituitaryーgonadal
LH: luteinizing hormone
OR: odds ratio
SD: standard deviations
SPL: stretched penile length
SRD5A2: steroid 5α‐reductase type 2 gene
TRT: testosterone replacement therapy
ΔGlans width: post‐treatment glans width minus baseline glans width
ΔSPL: post‐treatment SPL minus baseline SPL

## 1 Introduction

Micropenis is defined as a stretched penile length (SPL) below −2.5 standard deviations (SD) from the age‐specific reference, in the absence of anatomical anomalies such as hypospadias [1]. Since SPL norms vary across populations [2], age‐based Japanese reference values reported by Ishii et al. are commonly used in clinical practice [3]. Although the prevalence of micropenis is estimated to range between 0.015% and 0.3% in the general population [4, 5], it can exceed 20% among patients with conditions such as hypogonadotropic hypogonadism [6]. The etiology of micropenis includes not only hypogonadism but also disorders of androgen synthesis or action, growth hormone deficiency, and idiopathic or nonspecific causes [2].

Endocrine treatment of micropenis includes testosterone replacement therapy (TRT), dihydrotestosterone (DHT) cream or gel, and gonadotropin therapy. However, currently, DHT cream or gel is not approved for clinical use in Japan. Furthermore, data on the use of gonadotropin treatment in neonates and infants remain limited [2]. Additionally, the long‐term impact of these treatments on fertility remains insufficiently explored. Consequently, intramuscular injections of depot testosterone enanthate have become the standard therapy in Japan.

Several studies have addressed factors influencing the efficacy of TRT in pediatric patients with micropenis such as mutations in steroid 5α‐reductase type 2 gene (SRD5A2) and androgen receptor (AR), which have been identified as potential causes of poor response to TRT [7, 8]. However, previous studies have reported no significant correlations between TRT effectiveness and variables such as age, body surface area, or initial penile length [7]. Therefore, the present study retrospectively evaluated pediatric patients diagnosed with micropenis who received depot testosterone enanthate therapy. We aimed to investigate changes in SPL following TRT and identify clinical predictors associated with therapeutic response.

## 2 Methods

### 2.1 Patients and Study Design

This retrospective study included pediatric patients diagnosed with micropenis who received TRT at Nagasaki University Hospital between April 2019 and March 2024. Eligible patients were boys aged ≤ 3 years, whose guardians provided informed consent. SPL and glans width were measured using standardized techniques as previously described [9, 10]; glans width refers to the maximum transverse diameter of the glans, including the thickness of the prepuce. Furthermore, we have newly defined integrated exploratory indices combining SPL with glans width for this analysis: SPL × glans width, calculated as the product of SPL by glans width (mm^2^) and SPL/glans width, representing the ratio of SPL to glans width (unitless). TRT comprised three intramuscular injections of testosterone enanthate (25 mg) administered at monthly intervals [11, 12, 13, 14]. Treatment efficacy was assessed 3–6 months after the final dose. Patients were excluded if they did not meet the age criteria, had comorbidities such as hypospadias or bifid scrotum, or received a number of injections other than three. Based on the previous SPL SD scores [3] adjusted for age at the time of outcome evaluation, the patients were classified into three groups: Group 1 (good response: SPL ≥ +1.0 SD), Group 2 (intermediate response: −1.0 SD < SPL < +1.0 SD), and Group 3 (poor response: SPL ≤ −1.0 SD). We compared clinical variables among the three groups. Clinical variables included the following: birth weight, age, height, body weight, SPL, glans width, SPL × glans width, SPL/glans width at TRT initiation, presence of comorbidities, SPL and glans width at the outcome evaluation, ΔSPL (post‐treatment SPL minus baseline SPL), SPL growth ratio (GR), ΔGlans width (post‐treatment glans width minus baseline glans width), and glans width GR. In addition, patients were divided into two groups based on whether they started TRT during the mini‐puberty period (0–6 months of age) [15, 16] or not, and its association with the response groups was analyzed. Serum levels of luteinizing hormone (LH), follicle‐stimulating hormone (FSH), and testosterone at TRT initiation were dichotomized into “within the reference range” and “below the reference range” according to age‐based standards [17], and distributions were compared across the three response groups.

### 2.2 Data Collection

All clinical data were retrospectively collected from electronic medical records. Demographic and baseline characteristics, including age, height, weight, and comorbidities, were extracted. Measurements of SPL and glans width were performed by two pediatric urologists using the standardized methods within the same institution.

### 2.3 Outcomes

The primary outcome of this study was the post‐treatment increase in SPL, which was used to evaluate TRT response and identify clinical predictors of favorable outcomes. The secondary outcomes included the timing of treatment initiation in relation to the mini‐puberty period, as well as pre‐treatment hormone levels (LH, FSH, and testosterone).

### 2.4 Statistical Analysis

Categorical variables are expressed as percentages, and continuous variables were presented as means ± SD when normally distributed, or as medians with interquartile ranges (IQR) when non‐normally distributed. The Shapiro–Wilk test was used for assessing normality. If the distribution was normal, the Levene test was used for evaluating homogeneity of variance. Depending on the results, one‐way analysis of variance or Welch's test was applied. The Kruskal–Wallis test was used for non‐normal distributions. Fisher's exact test was used for categorical data comparisons.

To explore predictors of treatment response, logistic regression analyses were performed. Variables with significant differences from univariate analyses were fitted into multivariate analysis. Additionally, standardized logistic regression analysis using *z*‐scores was performed to assess the relative influence of each variable. All statistical analyses were performed using JMP Student Edition 18.2.0 for Windows (SAS Institute Inc., Cary, NC, USA), and statistical significance was set at *p* ≤ 0.05.

## 3 Results

A total of 37 boys were included in the study and classified into three groups based on treatment response (Table 1): Group 1 (*n* = 11), Group 2 (*n* = 17), and Group 3 (*n* = 9). Birth weight, height, body weight, SPL, glans width, and SPL × glans width were lower in Group 3; significant differences were found among the three groups (*p* = 0.049, 0.003, 0.001, 0.019, 0.020, and 0.001, respectively). On the other hand, no significant differences were found among the groups in terms of SPL/glans width (*p* = 0.401).

**TABLE 1:** Clinical characteristics of the patients at TRT initiation.

Variables		Overall	Group 1	Group 2	Group 3	
		(*n* = 37)	(*n* = 11)	(*n* = 17)	(*n* = 9)	*p*
Birth weight, g (Median [IQR])		3068 [2639–3305]	3164 [2618–3544]	3096 [2994–3347]	2660 [2162–2871]	0.049
Age, months (Median [IQR])		9.0 [6.0–11.0]	10.0 [6.0–11.0]	10.0 [6.0–12.5]	6.0 [5.0–9.0]	0.086
Height, cm (Median [IQR])		69.0 [65.6–73.6]	70.0 [68.0–73.2]	71.4 [66.2–79.7]	65.0[63.3–67.7]	0.003
Body weight, g (Median [IQR])		8380 [7390–9420]	9180 [8050–9870]	8380 [8037–10 775]	7165 [6830–7655]	0.001
SPL, mm (Median [IQR])		22.0 [20.0–25.0]	25.0 [22.0–27.0]	20.0 [20.0–23.5]	20.0 [20.0–22.8]	0.019
Glans width, mm (Median [IQR])		10.0 [9.0–10.3]	10.0 [10.0–12.0]	10.0 [8.0–10.5]	10.0 [9.0–10.0]	0.020
SPL × glans width, mm² (Median [IQR])		225 [185–250]	250 [250–270]	200 [160–240]	200 [180–228]	0.001
SPL/glans width (Median [IQR])		2.3 [2.0–2.5]	2.4 [2.0–2.5]	2.4 [2.1–2.6]	2.2 [2.1–2.3]	0.401
Comorbidities						
Low birth weight		6	2	1	3	
Undescended testis	Unilateral	3	1	1	1	
	Bilateral	2	1	1		
Kallmann syndrome		1			1	

Abbreviations: IQR, interquartile range; SPL, stretched penile length; TRT, testosterone replacement therapy.

At follow‐up (Table 2), the median SPL was 40.0, 35.0, and 28.0 mm in Groups 1, 2, and 3, respectively, indicating the most robust response in Group 1. Both ΔSPL and SPL GR were greater in Groups 1 and 2 compared with Group 3. However, intergroup differences were not found in glans width or glans width GR.

**TABLE 2:** Penile response in the patients after TRT.

Variables	Overall	Group 1	Group 2	Group 3
	(*n* = 37)	(*n* = 11)	(*n* = 17)	(*n* = 9)
SPL, mm (Median [IQR])	35.0 [31.0–40.0]	40.0 [40.0–45.0]	35.0 [35.0–37.5]	28.0 [27.8–30.0]
ΔSPL, mm (Median [IQR])	15.0 [9.0–15.5]	17.0 [15.0–20.0]	15.0 [12.0–15.0]	7.5 [6.3–9.0]
SPL GR, % (Median [IQR])	160 [140–175]	168 [150–200]	175 [155–175]	138 [128–145]
Glans width, mm (Median [IQR])	13.0 [13.0–14.0]	14.0 [13.5–14.0]	13.0 [13.0–14.0]	13.0 [12.0–13.0]
ΔGlans width, mm (Median [IQR])	3.0 [3.8–4.0]	3.0 [2.5–4.0]	3.5 [3.0–5.3]	3.0 [2.3–3.8]
Glans width GR, % (Median [IQR])	133 [125–144]	130 [125–140]	137 [128–163]	130 [123–141]

Abbreviations: GR, growth ratio; IQR, interquartile range; SPL, stretched penile length; TRT, testosterone replacement therapy; ΔGlans width, post‐treatment grans width minus baseline grans width. ΔSPL, post‐treatment SPL minus baseline SPL.

### 3.1 Timing of TRT Initiation During Mini‐Puberty (Table 1 and Figure 1)

**FIGURE 1:**
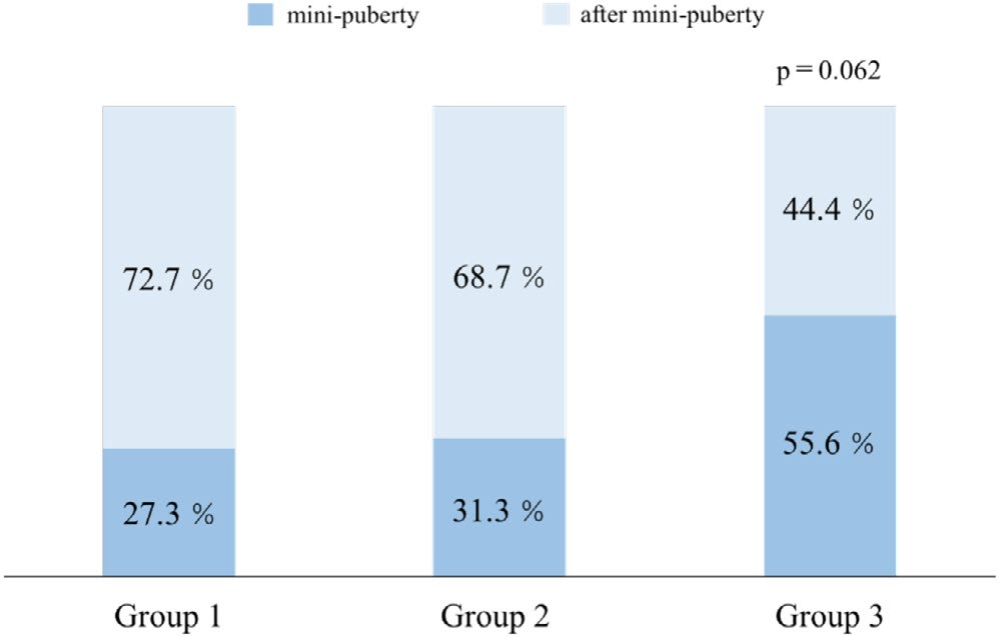
Proportion of patients who initiated TRT during mini‐puberty. Stacked bar graph showing the percentage of patients who began TRT during (0–6 months, dark blue) and after mini‐puberty (> 6 months, light blue) across response groups. Group 3 had the highest proportion of patients treated during mini‐puberty. TRT, testosterone replacement therapy.

The median age at TRT initiation was 10.0 months in Groups 1 and 2, and 6.0 months in Group 3. Although the patients in Group 3 initiated TRT at a younger age, the difference was not statistically significant (*p* = 0.086). The proportion of patients who began TRT during the mini‐puberty period was the highest in Group 3 (55.6%) compared with Groups 1 (27.3%) and 2 (31.3%).

### 3.2 Association With LH, FSH, and Testosterone Levels (Figure 2)

**FIGURE 2:**
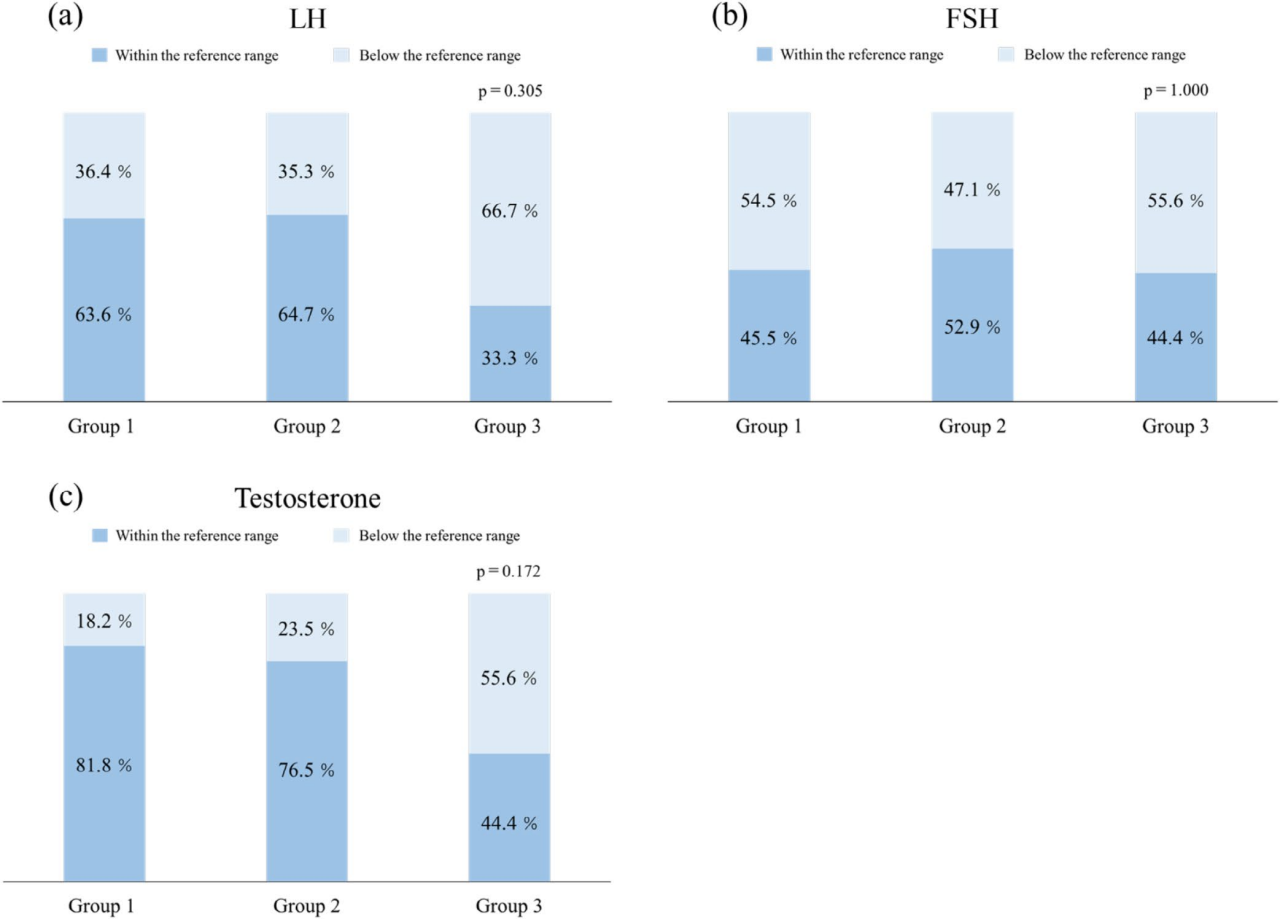
Proportions of patients with below the reference gonadotropin and testosterone levels across response groups. Stacked bar graphs showing the percentage of patients with serum LH (a), FSH (b), and testosterone (c) levels classified as below (dark blue) or within (light blue) the age‐specific reference range at the time of TRT initiation in Groups 1–3. No statistically significant differences were observed among the groups. LH, luteinizing hormone; FSH, follicle‐stimulating hormone; TRT, testosterone replacement therapy.

A higher proportion of patients in Group 3 exhibited below the reference levels of LH and testosterone; however, these differences were not statistically significant (*p* = 0.305, 1.000, and 0.172, respectively).

### 3.3 Predictors of TRT Response (Tables 3 and 4)

**TABLE 3:** Logistic regression analysis of clinical variables at TRT initiation for predicting TRT response.

Variables	Univariate	*p*	Multivariate	*p*
	OR (95% CI)		OR (95% CI)	
Birth weight, g	1.000 (0.999–1.002)	0.667		
Age, months	1.007 (0.879–1.133)	0.907		
Height, cm	1.022 (0.912–1.140)	0.694		
Body weight, g	1.000 (0.999–1.001)	0.463		
SPL, mm	1.367 (1.081–1.828)	0.008	1.315 (1.007–1.836)	0.044
Glans width, mm	2.304 (1.252–5.570)	0.005	2.249 (1.099–5.727)	0.024
SPL × glans width, mm²	1.029 (1.010–1.058)	0.001		
SPL/glans width	0.814 (0.105–5.733)	0.835		

Abbreviations: OR, odds ratio; CI, confidence interval; SPL, stretched penile length; TRT, testosterone replacement therapy.

**TABLE 4:** Standardized logistic regression analysis using *z*‐scores to SPL and glans width.

Variables	Standardized *β*	95% CI	*p*
SPL (*z*‐scores)	0.873	0.022–1.939	0.063
Glans width (*z*‐scores)	1.240	0.144–2.670	0.049

Abbreviations: CI, confidence interval; SPL, stretched penile length.

To identify predictors of favorable TRT response, we compared Group 1 with Groups 2 and 3 combined. Using univariate logistic regression analysis, baseline SPL (odds ratio [OR], 1.367; 95% confidence interval [CI], 1.081–1.828; *p* = 0.008), glans width (OR, 2.304; 95% CI, 1.252–5.570; *p* = 0.005), and SPL × glans width (OR, 1.029; 95% CI, 1.010–1.058; *p* = 0.001) were significant factors. Multivariate analysis showed that both baseline SPL (OR, 1.315; 95% CI, 1.007–1.836; *p* = 0.044) and glans width (OR, 2.249; 95% CI, 1.099–5.727; *p* = 0.024) remained significant independent predictors.

Due to moderate multicollinearity with SPL and glans width (variance inflation factor = 3.043 and 3.295), the SPL × glans width variable was excluded from the multivariate model. Standardized logistic regression analysis further indicated that glans width had a stronger predictive value than did SPL (standardized coefficient *β* = 1.240, *p* = 0.049).

## 4 Discussion

This study evaluated the clinical predictors associated with the efficacy of TRT in boys diagnosed with micropenis. In particular, we examined penile measurements at TRT initiation, the timing of TRT initiation in relation to the mini‐puberty period, and baseline levels of LH, FSH, and testosterone. Treatment response was assessed based on post‐treatment SPL and categorized into three groups using age‐specific SD scores. Our findings revealed significant intergroup differences in the initial penile parameters, including SPL, glans width, and SPL × glans width. In contrast, the median age at TRT initiation did not significantly differ among the groups; however, Group 3 initiated treatment earlier, with a higher proportion of patients starting during the mini‐puberty period. Additionally, a higher percentage of Group 3 patients had LH and testosterone levels below the reference range; however, these differences were not statistically significant. Using multivariate analysis, both baseline SPL and glans width were identified as independent predictors of TRT response, with glans width showing stronger predictive power using standardized logistic regression.

Previous studies have reported that the median SPL at TRT initiation in patients with micropenis is typically 2.0–2.5 cm, with post‐treatment SPL reaching 3.5–4.4 cm [7, 18]. Our findings are consistent with these values. Although changes in glans width following TRT have been scarcely reported in the context of micropenis, our data showed a median increase of 3.0 mm (133% increase) after three TRT sessions. Although glans width changes have been documented in patients with hypospadias (2.6–4.1 mm increase) [19, 20, 21], few studies have focused on patients with micropenis. Our findings revealed that both SPL and glans width were significant independent predictors of treatment response. It has been demonstrated that two physiological testosterone surges occur prenatally and during mini‐puberty, respectively, leading to androgen‐dependent penile growth during these periods [2, 22, 23, 24]. Therefore, SPL and glans width at TRT initiation might reflect the extent of prior androgen exposure and intrinsic androgen sensitivity of penile tissues. Since the therapeutic effect of TRT depends on the androgen responsiveness of penile tissues, patients with greater baseline SPL and glans width might have a higher potential for penile growth in response to TRT, making these parameters significant predictors of treatment efficacy. The previous literature has reported conflicting findings regarding correlations between initial penile length and TRT efficacy. Some studies found no association [7], whereas others reported strong effect sizes (*r* = 0.889 and 0.853) for SPL and penile width [18]. Our results indicated that both SPL and glans width might serve as useful clinical predictors of TRT efficacy in patients with micropenis. Since both are simple, noninvasive measurements, they may aid in treatment decision‐making and patient counseling. Although the composite index SPL × glans width was also significant using the univariate analysis, it was excluded from multivariate analysis due to moderate multicollinearity. Further studies are warranted to validate the clinical utility of such composite indicators.

Mini‐puberty is characterized by a transient elevation in gonadotropins and testosterone during early infancy and plays a crucial role in the maturation of Sertoli and Leydig cells as well as in the establishment of future fertility potential [16]. Several studies have suggested that gonadotropin supplementation during this period may promote testicular development and descent [25, 26]. However, its relevance to the management of micropenis remains uncertain. An intriguing finding from our study is that initiating TRT during mini‐puberty did not necessarily result in favorable outcomes. Although it is traditionally assumed that aligning TRT with the physiological rise in endogenous testosterone during mini‐puberty might produce more physiological and thus more effective results, our data contradict this assumption. Group 3, which included the highest proportion of patients who began TRT during mini‐puberty, showed the poorest penile response. Although supplementing exogenous testosterone in parallel with the endogenous hormonal surge of mini‐puberty might appear biologically reasonable, our findings suggest that increasing testosterone levels beyond the physiological peak may not enhance penile growth. Rather, initiating TRT after the natural decline in testosterone following mini‐puberty might be more beneficial. This could provide a stronger anabolic stimulus at a time when endogenous androgen levels are otherwise low. Androgen action is known to plateau once circulating testosterone levels exceed the binding capacity threshold of ARs [27]. This saturation effect may limit the additional benefit of further testosterone elevation during mini‐puberty. However, our study found no significant correlation between the degree of penile growth and timing of TRT initiation. Validating this hypothesis requires further investigation. Although the percentage of patients with subnormal LH and testosterone levels was higher in Group 3, no statistically significant differences were observed among the three groups. Furthermore, none of the patients exhibited abnormally elevated hormone levels. Among the 13 patients who initiated TRT during mini‐puberty, four exhibited subnormal testosterone levels. These findings suggest that even during mini‐puberty, the endogenous secretion of testosterone may vary considerably among individuals. Following TRT, three of the four patients with initially low testosterone levels showed a sufficient increase in SPL to be classified into the good‐response group. Subnormal gonadotropin and testosterone levels during early infancy have been proposed as potential indicators of hypothalamicーpituitaryーgonadal (HPG) axis dysfunction later in childhood [6, 28]. The relatively high proportion of patients with low LH and testosterone levels in Group 3—the group that exhibited the poorest response to TRT—suggests that further evaluation of HPG axis function might be warranted in patients whose SPL remains below −1.0 SD after treatment. Notably, our dataset also included a patient diagnosed with Kallmann syndrome within this group. Highlighting that hormone levels in this study were assessed at only a single time point prior to TRT is important, which may not accurately reflect overall HPG axis activity or account for diurnal variations. Future investigations should incorporate more comprehensive hormonal assessments, including stimulation tests and time‐controlled sampling, to better evaluate the functional status of the HPG axis.

This study has some limitations that should be considered. As a retrospective, single‐center investigation, it may be subject to selection and information biases, which could limit the generalizability of the results. The relatively small sample size in each response group might have reduced the statistical power to detect subtle associations. Hormone levels were assessed only once prior to treatment, without accounting for diurnal variation or performing a gonadotropin‐releasing hormone stimulation test, potentially affecting the accuracy of endocrine evaluation. In addition, genetic testing for SRD5A2 and AR abnormalities was not conducted, and with the exception of one patient diagnosed with Kallmann syndrome, the primary cause remained undetermined. Future prospective multicenter studies incorporating serial hormonal assessments and genetic analyses are warranted to validate and extend these findings.

## Author Contributions

**Junki Harada:** conceptualization, data curation, formal analysis, investigation, writing – original draft, methodology, visualization, software. **Kensuke Mitsunari:** conceptualization, formal analysis, validation, methodology, writing – original draft, project administration. **Shota Kakita:** data curation. **Hajime Fukushima:** data curation. **Haruka Kawamura:** data curation. **Hiroki Kurata:** data curation. **Tsuyoshi Matsuda:** investigation. **Midori Motokawa:** data curation. **Itsuho Ito:** investigation. **Kyohei Araki:** investigation. **Yuichiro Nakamura:** investigation. **Tomohiro Matsuo:** formal analysis. **Sumito Dateki:** supervision. **Toshiharu Kihara:** data curation. **Kojiro Ohba:** writing – review and editing. **Yasushi Mochizuki:** writing – review and editing. **Ryoichi Imamura:** supervision.

## Consent

Informed consent was obtained from the patients' guardians through an opt‐out method described on the hospital website.

## Conflicts of Interest

The authors declare no conflicts of interest.
